# Objective evaluation of hand physiotherapy in juvenile systemic sclerosis using the MediTutor robotic glove

**DOI:** 10.1186/s12969-026-01237-0

**Published:** 2026-06-18

**Authors:** Natalie Kleistnerova, Michal Tomcik, Maja Spiritovic

**Affiliations:** 1 Department of Health Care and Population Protection, Faculty of Biomedical Engineering, Czech Technical University in Prague (CTU FBMI), Prague, Czech Republic; 2 Institute of Rheumatology, Prague, Czech Republic

**Keywords:** Juvenile systemic sclerosis, Fibrosis, Pediatric rheumatology, Fine motor skills, Hand function, Physiotherapy, MediTutor, HandTutor, Therapy putty

**Dear Editor**,

Juvenile systemic sclerosis (JSSc) is a rare condition associated with progressive fibrosis leading to impaired hand mobility and function, requiring early targeted intervention [1]. However, objective monitoring of hand physiotherapy outcomes in pediatric rheumatology remains limited [2].

The robotic glove represents an innovative tool enabling standardized, quantitative assessment of hand movement and functional capacity. In this context, we demonstrate its clinical applicability during a six-month physiotherapy program in a pediatric JSSc case.

A 12-year-old patient with JSSc underwent a structured physiotherapy program (biweekly sessions over six months) combining thermotherapy, myofascial techniques, joint mobilization, and active hand training including fine motor exercises using TherapyPutty [3, 4].

The HandTutor robotic glove (CE-certified and FDA-approved) [5, 6] was used for objective assessment of hand function as a wearable device enabling real-time measurement. It operates on the principle of integrated biofeedback, with embedded sensors capturing finger and wrist movements and translating kinematic parameters (e.g., range. of motion, movement speed, and coordination) into quantitative digital outputs. This enables standardized, reproducible, and clinically relevant evaluation of both active and passive movement. The recorded data in our patient with JSSc demonstrated increased range of motion across most fingers and both wrists, with the greatest improvement observed in passive movement of the right thumb and left middle finger (+ 5 mm).

These instrument-based findings were consistent with clinical outcomes assessed using standard methods: grip strength measured with a digital dynamometer increased (R: +5.76 N; L: +17.93 N), the modified Rodnan Skin Score decreased (11→9), the finger-to-palm distance improved by 1 cm bilaterally, and the Hand Mobility in Scleroderma test showed improvement in finger flexion and pinch grip. The patient also subjectively reported reduced hand stiffness and resolution of wrist pain during loading (Fig. 1).

**Fig. 1:**
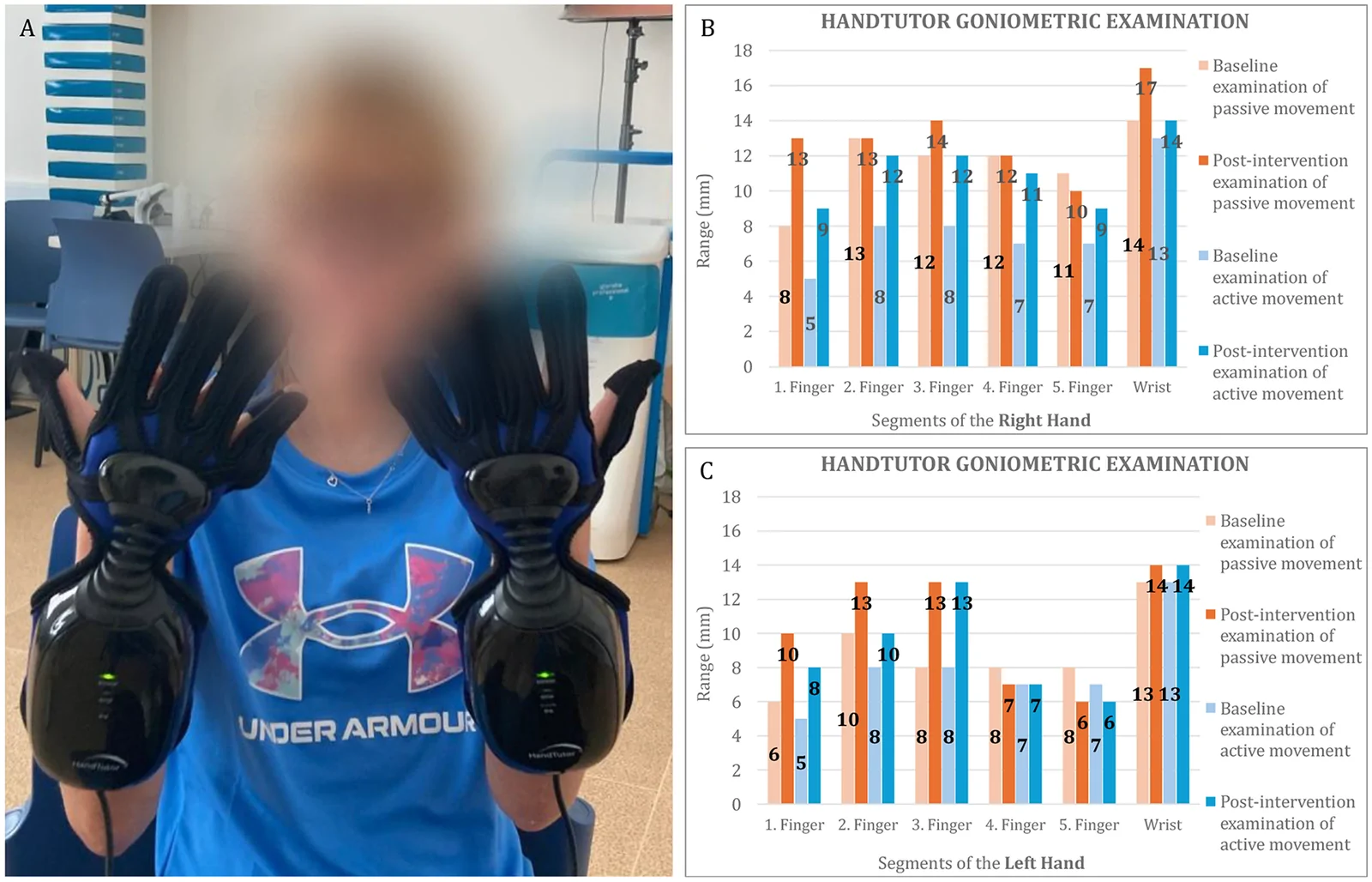
Objective evaluation of hand function using the MediTutor system; **A** The HandTutor robotic glove during objective goniometric assessment. **B–C** Baseline versus post-intervention comparison of active and passive range of motion (mm) for the right (**B**) and left (**C**) hand segments

This case illustrates how the integration of advanced evaluation technologies such as the HandTutor robotic glove into targeted physiotherapy can enhance clinical effectiveness and complement systemic therapy in JSSc [7]. Unlike traditional goniometry, which is dependent on examiner experience, this device enables precise kinematic tracking of both active and passive range of motion in finger joints and the wrist. Its interactive graphical interface may also support patient engagement and motivation. However, the device is not only used for assessment and measurement but can also be applied therapeutically; this capability was not utilized in the present study. 

We suggest that the incorporation of objective, technology-based assessment tools like HandTutor can significantly improve the monitoring and management of fine motor impairments in JSSc. Importantly, although not investigated in this study, the system has the potential to support home-based use and remote monitoring through its dedicated software platform, thereby facilitating telerehabilitation and continuity of care beyond the clinical setting.

Beyond JSSc, the clinical applicability of this robotic glove extends to a wide range of hand pathologies, including inflammatory conditions such as Juvenile Idiopathic Arthritis (JIA) and non-inflammatory impairments such as post-traumatic contractures, congenital hand anomalies, or neurological deficits (e.g., hemiparesis). From a practical perspective, the device is feasible for routine clinical use due to its relatively low maintenance requirements, ease of implementation, and availability in adult sizes, allowing direct translation into adult clinical practice. 

In addition, the global availability and cost-effectiveness of this device highlight its practical applicability in routine clinical settings.

## Data Availability

No datasets were generated or analysed during the current study.

## Declarations

The authors certify that they have obtained all appropriate patient consent forms.
